# Maximum type I error rate inflation from sample size reassessment when investigators are blind to treatment labels

**DOI:** 10.1002/sim.6848

**Published:** 2015-12-23

**Authors:** Magdalena Żebrowska, Martin Posch, Dominic Magirr

**Affiliations:** ^1^ Center for Medical Statistics, Informatics and Intelligent SystemsMedical University of ViennaVienna1090Austria

**Keywords:** sample size reassessment, type I error rate control, adaptive clinical trials, random allocation, block randomization, blinded interim analysis

## Abstract

Consider a parallel group trial for the comparison of an experimental treatment to a control, where the second‐stage sample size may depend on the blinded primary endpoint data as well as on additional blinded data from a secondary endpoint. For the setting of normally distributed endpoints, we demonstrate that this may lead to an inflation of the type I error rate if the null hypothesis holds for the primary but not the secondary endpoint.

We derive upper bounds for the inflation of the type I error rate, both for trials that employ random allocation and for those that use block randomization. We illustrate the worst‐case sample size reassessment rule in a case study. For both randomization strategies, the maximum type I error rate increases with the effect size in the secondary endpoint and the correlation between endpoints. The maximum inflation increases with smaller block sizes if information on the block size is used in the reassessment rule. Based on our findings, we do not question the well‐established use of blinded sample size reassessment methods with nuisance parameter estimates computed from the blinded interim data of the primary endpoint. However, we demonstrate that the type I error rate control of these methods relies on the application of specific, binding, pre‐planned and fully algorithmic sample size reassessment rules and does not extend to general or unplanned sample size adjustments based on blinded data. © 2015 The Authors. *Statistics in Medicine* Published by John Wiley & Sons Ltd.

## 1 Introduction

Blinding is a generally accepted tool to address bias in randomized clinical trials. It ensures that up to the investigated intervention all subjects are handled equally across treatment groups and outcomes are assessed in the same way. Furthermore, blinding of study subjects allows one to distinguish specific treatment effects from potential placebo effects. Blinding is also essential to avert statistical bias in hypotheses testing procedures if data dependent changes to the analysis strategy are made. The ICH E9 guideline 1, for example, recommends to review (and possibly update) the statistical analysis plan based on a blinded data review and notes that “Decisions made at this time should be described in the report, and should be distinguished from those made after the statistician has had access to the treatment codes, as blind decisions will generally introduce less potential for bias”. Similarly, in adaptive clinical trials where adaptations of the trial designs such as a reassessment of sample size can be performed after an interim analysis, blinding is important: it is well known that sample size reassessment based on unblinded interim data may lead to inflation of the type I error by more than 100% 2, 3 if the adaptation is not accounted for by using appropriate adaptive testing procedures 4, 5, 6. To address the various sources of bias in adaptive trials, regulatory guidelines 7, 8, 9 recommend to avoid breaking the blind and to perform adaptations based on blinded interim analyses instead. An assumption underlying these guidance documents is that adaptations based on blinded interim analyses are less prone to bias. Indeed, it has been demonstrated in several settings that adaptations based on blinded interim analysis do not require an adjusted analysis to control the type I error:

- for superiority studies comparing normally distributed endpoints in a parallel group design where the sample size is reassessed based on the “lumped variance” (the variance of the total sample pooling the observations from both groups), the type I error rate is essentially unaffected 10, 11, 12),
- also for superiority studies comparing event rates, where the sample size is reassessed based on the overall number of events across treatment groups, no relevant inflation of the type I error rate was observed 13. Analogous results were obtained also for count data 14,
- if permutation tests are applied, Posch & Proschan 15 and Proschan *et al*. 16 showed that adaptations based on blinded interim data will indeed control the type I error rate if the clinical trial is restricted to a univariate testing problem where a single endpoint is observed. If adaptations are restricted to the choice between endpoints, the result extends to trials where two endpoints are considered simultaneously. Asymptotically, these results are also valid for *t*‐tests.

However, blinding is not a panacea to prevent bias. If sample sizes are low, a minor increase of the type I error rate is observed for non‐inferiority trials with sample size reassessment based on the lumped variance 17. Also in superiority tests, Proschan *et al*. 16 showed that for general sample size reassessment rules based on the lumped variance the type I error rate may be inflated for small sample sizes. Furthermore, if the sample size reassessment rule may depend on more than one endpoint, type I error rate control is no longer guaranteed: if the null hypothesis holds for the primary endpoint but not for a secondary endpoint such as, for example, the level of drug in the blood, the secondary endpoint may completely unblind the investigator. However, the bias can also occur in less extreme settings, where the secondary endpoint unblinds the investigator only partially, as may be the case for a safety endpoint. In such settings, the potential type I error rate inflation is similar to that of a clinical trial where adaptations are performed in an unblinded interim analysis without being accounted for in the testing strategy 15.

In this paper, we investigate the potential consequences of blinded sample size reassessment approaches that deviate from the accepted statistical practice of applying a binding, algorithmic, and blinded sample size reassessment procedure for which type I error rate control has been demonstrated. In particular, we consider settings where no blinded sample size reassessment has been pre‐specified in the protocol, settings where an option for blinded sample size reassessment (but no binding rule) are pre‐specified, and settings where a binding rule have been pre‐specified but the data monitoring committee decided not to follow the rule. Sponsors may argue for a more flexible approach for several reasons: for example, the deviation of nuisance parameter estimates from planning assumptions may not have been anticipated in the planning phase; the maximum number of available patients is unknown in advance such that no binding rule can be pre‐specified; recruitment is lower than anticipated or safety concerns arise such that it is argued that the pre‐planned sample size algorithm cannot be followed; or information from other trials may arise that serves as an argument for a change in pre‐specified strategies. Recent regulatory guidance documents appear to acknowledge such unplanned adaptations. For example, the FDA adaptive designs draft guidances state, “Certain blinded‐analysis‐based changes, such as sample size revisions based on aggregate event rates or variance of the endpoint, are advisable procedures that can be considered and planned at the protocol design stage, but can also be applied when not planned from the study outset if the study has remained unequivocally blinded.” 8 and “While it is strongly preferred that such adaptations be preplanned at the start of the study, it may be possible to make changes during the studyŠs conduct as well. In such instances, the FDA will expect sponsors to be able to both justify the scientific rationale why such an approach is appropriate and preferable, and demonstrate that they have not had access to any unblinded data (either by coded treatment groups or completely unblinded) and that the data has been scrupulously safeguarded.” 9. Unplanned sample size adjustment is also accepted by European regulators in specific settings, see, for example, Case Study 3 in 18.

We consider the setting of a superiority test of a new experimental treatment over control, with a parallel group design and both blocked and unblocked randomization, where the sample size is reassessed after a blinded interim analysis. We assume that blinded data of the primary and a secondary endpoint is observed. This secondary endpoint – which may or may not be correlated with the primary endpoint – could be a surrogate endpoint, a clinical outcome, or a biomarker. For simplicity, the joint distribution of the two endpoints is assumed to be bivariate normal. If the null hypothesis of no treatment effect holds for the primary but not for the secondary endpoint, then the blinded secondary endpoint data provide the investigator with some information about the likely treatment assignment. We quantify the extent to which this can lead to biased analysis results.

In Section 2, we introduce the notation and statistical model. In Section 3, we derive an upper bound on the type I error rate for a trial using a random allocation strategy. The case of blocked randomization is considered in Section 4.

The results are applied to a case study in Section 5, and the impact of our investigation on the conduct of blinded interim analyses in clinical trials is discussed in Section 6.

## 2 The model

Consider a parallel group comparison of an experimental treatment to a control with *n* subjects in total. Denote the primary endpoint measurement of subject *i* = 1,…,*n* by *X*_*i*_, and let *G*_*i*_∈{0,1} denote the random treatment allocation. We assume that outcomes are normally distributed with means $\mu_{G_{i}}$ and common variance *σ* ^2^. A hypothesis test of

$$H_{0}:\mu_{1}\leq \mu_{0}\text{against}H_{1}:\mu_{1}>\mu_{0}$$

is performed at level *α*. After *n*_1_=*n*/2 observations, an interim analysis is performed, and the sample size is reassessed. The new second‐stage sample size is denoted by *n*_2_, and the new total sample size is *N*:=*n*_1_+*n*_2_. Besides the primary endpoint *X*_*i*_, we assume that the experimenter also observes a secondary endpoint *Y*_*i*_ for each subject *i*. Assume that the response of patient *i*, conditional on *G*_*i*_, is distributed as

$$\left(\begin{array}{c} X_{i}\\ Y_{i} \end{array}\right)|G_{i}=g_{i}∼N\left(\left(\begin{array}{c} 0\\ g_{i}\nu_{1}+(1-g_{i})\nu_{0} \end{array}\right),\sigma^{2}\left(\begin{array}{cc} 1 & \rho \\ \rho & 1 \end{array}\right)\right)\;,$$

where *ν*_1_,*ν*_0_ are the means of the secondary endpoint in treatment and control groups, respectively. At the end of the trial, *H*_0_ will be rejected if *Z*_*N*_>*z*_1 − *α*_ where, assuming balanced group sizes,

$$\mathrm{rCl}\begin{array}{ccc} Z_{N} & = & \frac{1}{\sigma \sqrt{N}}\overset{N}{\underset{i=1}{\sum }}\left\{1(G_{i}=1)-1(G_{i}=0)\right\}X_{i}=\frac{1}{\sigma \sqrt{N}}\overset{N}{\underset{i=1}{\sum }}(2G_{i}-1)X_{i}. \end{array}$$

The maximum conditional type I error rate given the first stage data from both endpoints is therefore

(1) $$\underset{n_{2}\in (0,\infty )}{\max}P\left\{Z_{N}>z_{1-\alpha }|(X_{i},Y_{i}){}_{i=1}^{n_{1}}\right\}.$$

## 3 Random allocation

Our aim is to quantify the extent of potential type I error rate inflation when the outcomes of a secondary endpoint provide partial information about the treatment assignment. We first wish to quantify this inflation for a “random allocation” strategy, where exactly *n*_1_/2 patients received the experimental treatment, with each of the

(2) $$\frac{n_{1}!}{(n_{1}/2)!(n_{1}/2)!}$$

possible sequences of $G=(G_{1},G_{2},\ldots ,G_{n_{1}})$ equally likely. After *n*_1_ responses have been observed, a blinded interim analysis is performed, and a second stage sample size *n*_2_ is chosen. We further assume random allocation in the second stage such that exactly *n*_2_/2 patients receive the experimental treatment. The maximum conditional type I error rate given the first stage data from both endpoints is given by (1). Exact evaluation of (1) is difficult for all, but the smallest *n*_1_ because the number of possible assignments (2) grows exponentially. We approach this problem in two ways. Firstly, we use an MCMC algorithm to simulate from the conditional distribution of ***G*** given the blinded interim data. Secondly, we derive asymptotic results for a “simple randomization” strategy – that is, assuming each patient is allocated to the experimental treatment independently with probability 1/2 – and use a combination of heuristic arguments and simulation results to show that the same asymptotic results are applicable to random allocation.

### 3.1 Computational approach

The type I error rate conditional on the unblinded data $(X_{i},Y_{i},G_{i}){}_{i=1}^{n_{1}}$ is easy to compute:

(3) $$\begin{array}{cc} P\left\{Z_{N}>z_{1-\alpha }|(X_{i},Y_{i},G_{i}){}_{i=1}^{n_{1}}\right\}= & \;P\left\{\frac{1}{\sigma \sqrt{n_{2}}}\overset{N}{\underset{i=n_{1}+1}{\sum }}(2G_{i}-1)X_{i}>\sqrt{\frac{N}{n_{2}}}z_{1-\alpha }-\sqrt{\frac{n_{1}}{n_{2}}}Z_{1}\right\}\\ = & \;1-\Phi \left(\sqrt{\frac{N}{n_{2}}}z_{1-\alpha }-\sqrt{\frac{n_{1}}{n_{2}}}Z_{1}\right), \end{array}$$

where $Z_{1}=\overset{n_{1}}{\underset{i=1}{\sum }}(2G_{i}-1)X_{i}/(\sigma \sqrt{n_{1}})$. It is therefore straightforward to find

(4) $$\mathrm{rCl}\begin{array}{ccc} P\left\{Z_{N}>z_{1-\alpha }|(X_{i},Y_{i}){}_{i=1}^{n_{1}}\right\} & = & \int P\left\{Z_{N}>z_{1-\alpha }|(X_{i},Y_{i},G_{i}){}_{i=1}^{n_{1}}\right\}\;dP\left\{(G)\overset{n_{1}}{\underset{i=1}{}}|(X_{i},Y_{i}){}_{i=1}^{n_{1}}\right\} \end{array}$$

provided that we can integrate over the conditional distribution of ***G*** given the blinded data. Although this distribution is over a large space of possible permutations, it can be sampled from using standard MCMC techniques 19. To maximize this conditional type I error rate, we select the *N* that maximizes (4). R code is provided in the Supporting Information.

### 3.2 Asymptotic considerations and an upper bound for the type I error rate

While the aforementioned computational approach can tell us the maximum conditional type I error rate given a specific blinded data set $(x_{i},y_{i}){}_{i=1}^{n_{1}}$, it cannot tell us the overall properties of the sample size reassessment procedure without considerable computational effort. Therefore, we study the asymptotic conditional distribution of *Z*_1_. We first derive the conditional distribution of *Z*_1_ under simple randomization (instead of random allocation with fixed per group sample sizes) and then, based on heuristic arguments and supported by simulation, we argue that the same asymptotic distribution applies also for random allocation. If each patient is allocated to the experimental treatment independently with probability 1/2 then by Bayes' theorem

(5) $$\begin{array}{cc} P\left\{G_{j}=1|(X_{i},Y_{i}){}_{i=1}^{n_{1}}=(x_{i},y_{i}){}_{i=1}^{n_{1}}\right\}= & \;P\left\{G_{j}=1|(X_{j},Y_{j})=(x_{j},y_{j})\right\}\\ = & \;\frac{\varphi_{\nu_{1},\sigma ,\rho }(x_{j},y_{j})}{\varphi_{\nu_{0},\sigma ,\rho }(x_{j},y_{j})+\varphi_{\nu_{1},\sigma ,\rho }(x_{j},y_{j})}=:q_{j} \end{array}$$

for *j* = 1,…,*n*_1_, where $\varphi_{\nu_{1},\sigma ,\rho }(\cdot ,\cdot )$ and $\varphi_{\nu_{0},\sigma ,\rho }(\cdot ,\cdot )$ denote the density functions of the two dimensional normal distribution of (*X*_*j*_,*Y*_*j*_) under experimental treatment and control, respectively. By the central limit theorem for the sum of independent but non‐identically distributed random variables (e.g., Theorem 2.7.1 in 20),

$$Z_{1}|(X_{i},Y_{i}){}_{i=1}^{n_{1}}=(x_{i},y_{i}){}_{i=1}^{n_{1}}$$

is asymptotically normal with mean $m_{1}=\overset{n_{1}}{\underset{i=1}{\sum }}(2q_{i}-1)x_{i}/(\sigma \sqrt{n_{1}})$ and variance $V_{1}=4(\sigma^{2}n_{1}){}^{-1}\overset{n_{1}}{\underset{i=1}{\sum }}x_{i}^{2}q_{i}(1-q_{i})$. We argue that this approximation is valid also under random allocation as, for *n*_1_ large enough, the information provided by the known allocation ratio becomes negligible. In particular,

$$E\left\{G_{j}|(X_{i},Y_{i}){}_{i=1}^{n_{1}}=(x_{i},y_{i}){}_{i=1}^{n_{1}}\right\}\approx q_{j},\;\text{cov}\left\{G_{j},G_{k}|(X_{i},Y_{i}){}_{i=1}^{n_{1}}=(x_{i},y_{i}){}_{i=1}^{n_{1}}\right\}\approx 0\text{for}j\neq k.$$

**Figure 1:**
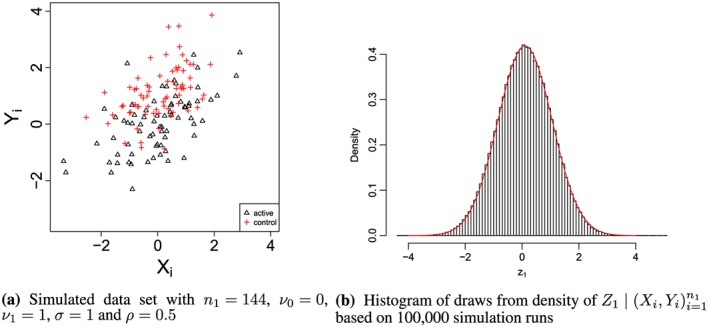
Comparing the asymptotic results with MCMC output for an example data set.

To add support to our claim, we simulated multiple data sets under various choices of *n*_1_,*ν*_1_ and *ρ*, and compared the normal approximation with the output of the MCMC algorithm. An example is shown in Figure 1, where the normal curve agrees well. As *ν*_1_ and *ρ* increase, it becomes easier to identify the likely treatment assignment, making the conditional distribution of *Z*_1_ more discrete and the speed of convergence to a normal distribution slower. This can be seen in Figure 9.1–9.3 of the supporting information. For a sample size of *n*_1_=144, the approximation appears to be adequate provided that *ν*_1_≤2 and *ρ*≤0.8.

Equation (5) is also useful to illustrate the impact of the secondary endpoint effect size *ν*_1_−*ν*_0_ on the potential to unblind the data: If *ν*_0_=*ν*_1_, then $q_{j}=\frac{1}{2}$ and the secondary endpoint gives no information on the treatment allocation. If, in contrast, |*ν*_1_−*ν*_0_| increases then *q*_*j*_(*X*,*Y*) converges in distribution either to 0 (for observations in the control) or to 1 (for observations from the experimental treatment group) even if the correlation *ρ* is zero: Indeed, for *ρ* = 0 and if *Y* is drawn, for example, from the control group then for all *ε* > 0, we have *P*(|*Y* − *ν*_0_|>*c*)≤*ε* for *c* large enough. However, for *y*, such that |*y* − *ν*_0_|≤*c*, we have $q(x,y)=\varphi_{\nu_{1},\sigma }(y)/\left[\varphi_{\nu_{0},\sigma }(y)+\varphi_{\nu_{1},\sigma }(y)\right]=1/\{1+\exp\left[(\nu_{1}-\nu_{0})(\nu_{1}+\nu_{0}-2y)/2\right]\}\to 0$ as |*ν*_1_−*ν*_0_|→*∞*.

To maximize the overall conditional error rate, note that for any given blinded first‐stage data set the maximum conditional type I error rate is

(6) $$\begin{array}{cc} \underset{n_{2}\in (0,\infty )}{\max}P\left\{Z_{N}>z_{1-\alpha }|(X_{i},Y_{i}){}_{i=1}^{n_{1}}=(x_{i},y_{i}){}_{i=1}^{n_{1}}\right\}\\ = & \underset{n_{2}\in (0,\infty )}{\max}P\left\{\frac{1}{\sigma \sqrt{N}}\overset{N}{\underset{i=n_{1}+1}{\sum }}(2G_{i}-1)X_{i}+\sqrt{\frac{n_{1}}{N}}Z_{1}>z_{1-\alpha }|(X_{i},Y_{i}){}_{i=1}^{n_{1}}=(x_{i},y_{i}){}_{i=1}^{n_{1}}\right\}\\ \approx & \underset{n_{2}\in (0,\infty )}{\max}1-\Phi \left(\frac{z_{1-\alpha }-\sqrt{\frac{n_{1}}{N}}m_{1}}{\sqrt{\frac{n_{1}V_{1}+n_{2}}{N}}}\right). \end{array}$$

Here, we approximated the conditional distribution of *Z*_1_ by a *N*(*m*_1_,*V*_1_) distribution. Assume there are minimum and maximum sample sizes $n_{2}^{\min},n_{2}^{\max}$ for the second stage sample size such that $n_{2}\in [n_{2}^{\min},n_{2}^{\max}]$. Then, the value of *n*_2_ maximizing (6) is (Appendix A)

(7) $${ñ}_{2}(m_{1},V_{1})=\left\{\begin{array}{cc} n_{2}^{\max} & \text{if}m_{1}<z_{*}\\ \left[\left(\frac{z_{1-\alpha }(1-V_{1})}{m_{1}}\right){}^{2}-1\right]n_{1} & \text{if}m_{1}\in \left[z_{*},z^{*}\right]\\ n_{2}^{\min} & \text{if}m_{1}>z^{*} \end{array}\right.$$

if *V*_1_≤1, and

(8) $${ñ}_{2}(m_{1},V_{1})=\left\{\begin{array}{cc} n_{2}^{\min} & \text{if}m_{1}>z^{*}\\ \left[\left(\frac{z_{1-\alpha }(1-V_{1})}{m_{1}}\right){}^{2}-1\right]n_{1} & \text{if}m_{1}\leq z^{*} \end{array}\right.$$

if *V*_1_>1, where $z_{*}=\frac{z_{1-\alpha }(1-V_{1})}{\sqrt{1+n_{2}^{\max}/n_{1}}},z^{*}=\frac{z_{1-\alpha }(1-V_{1})}{\sqrt{1+n_{2}^{\min}/n_{1}}}$.

**Figure 2:**
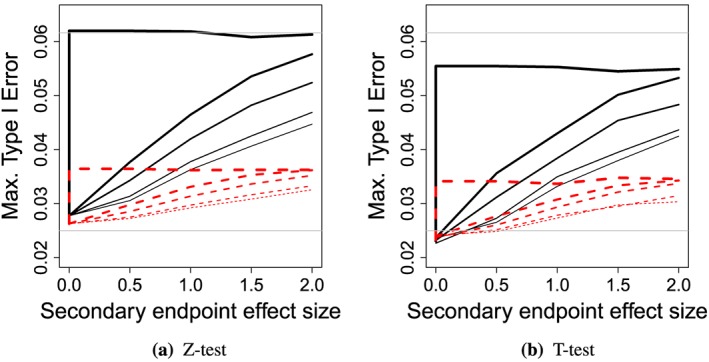
Maximum type I error rate as a function of the secondary endpoint effect size with and without restrictions for the second stage sample size. Here, the first stage sample size *n*_1_=144 and *σ* = 1. (Black) solid lines denote unrestricted results, and (red) dashed lines results for restricted case with $n_{2}^{\min}=n_{1}/2$ and $n_{2}^{\max}=4n_{1}$. *ρ*∈{0,0.5,0.8,0.9,1} and the larger the *ρ* the thicker the line.

Figure 2 shows the maximum type I error rate as function of the secondary endpoint effect size for different correlations between the primary and the secondary endpoint *ρ*∈{0,0.5,0.8,0.9,1}. The worst case conditional error rate was determined by simulation (200,000 simulation runs if not indicated otherwise) setting *σ* = 1, a nominal one‐sided significance level of 2.5*%*, and *n*_1_=144 (which is half the total sample size required for a *z*‐test with power 80*%* to detect an absolute treatment effect of 1/3 in the primary endpoint). We consider effect sizes in the secondary endpoint ranging from 0 to 2. On first sight, the latter may appear large for trials with the chosen sample size; however, effects in secondary or safety endpoints (such as, for example, laboratory parameters) are typically not relevant for the power calculation and may substantially differ from the treatment effect in the primary endpoint the study is powered for.

In each simulation run, primary and secondary endpoint data were simulated from a bivariate normal distribution, and the maximum conditional error rate was computed based on the approximation (6) with the second stage sample size as defined by (7) and (8). The final maximum type I error rate was then calculated based on the total *Z*‐test statistics *Z*_*N*_ for the unrestricted case with $n_{2}^{\min}=0$ and $n_{2}^{\max}=\infty$ and for the restricted case with $n_{2}^{\min}=n_{1}/2$ and $n_{2}^{\max}=4n_{1}$ Figure 2(a). For both cases, the maximum type I error rate increases with the correlation *ρ* between the primary and the secondary endpoint. If this correlation is *ρ* = 1, and providing that a secondary endpoint effect is present, the maximum type I error rate under unrestricted case is *α*_*max*_=0.062, which equals the maximum type I error rate inflation for an unblinded analysis reported by 2.

Careful examination of Figure 2(a) reveals that the type I error rate is already inflated when the secondary endpoint effect size is zero. This is an artifact of assuming the variance *σ* ^2^ to be known. In this case, *m*_1_=0 and *V*_1_ is proportional to $\sum \overset{2}{\underset{i}{x}}$. Rules (7) and (8) reduce to choosing *n*_2_ as small as possible when *V*_1_>1, that is, when there is excess variation in the blinded data, and as large as possible when *V*_1_<1. Intuitively, this is because a larger variance increases the chance of obtaining a significant result. In an attempt to remove this artifact, we re‐ran the simulations, but this time performing a *t*‐test at the final analysis Figure 2(b). In this case, the procedure becomes slightly conservative at a secondary endpoint effect size of zero. Again, this makes sense for a reassessment procedure that tends to produce a high variance estimate (since this term appears in the denominator of the t statistic).

## 4 Block randomization

Often, randomization is performed in blocks to guarantee that the treatment allocation frequencies in the earlier and later phases of a trial are balanced (e.g., Miller *et al.* (2009) 21). Consider a trial with block randomization with blocks of length *τ*, where *τ* is even and the treatment allocation is balanced (*P*(*G*_*i*_=0) = *P*(*G*_*i*_=1) = 1/2). In this section, we investigate the extent to which the additional information on the treatment allocation provided by the blocking allows one to introduce additional bias by sample size reassessment.

For example, for a block size of *τ* = 2, for each block, there are only two possible allocation sequences, *AB* and *BA*. Both have probability 1/2. Obviously, the conditional probability, given the blinded data, that the first subject has been assigned to group *A* is equal to the conditional probability of the allocation sequence *AB*, conditional on the data of the primary and secondary endpoints of both subjects in the block. As we now use data from two patients to estimate probability of the allocation sequence *AB* (which is the allocation probability of the first subject) and there are only two possible sequences, we obtain a more informative estimate than in the random allocation scenario, where the allocation probability of each patient was estimated based on its own data only. However, the additional information on allocation probabilities provided by the consideration of the allocation sequences decreases with the block size. For a block size of four, for example, there are 42=6 possible allocation sequences: *A* *A* *B* *B*,*A* *B* *A* *B*,*A* *B* *B* *A*,*B* *A* *B* *A*,*B* *B* *A* *A*,*B* *A* *A* *B*. Each has (unconditional) probability 1/6. To compute the conditional probability that the first patient is in group *A*, given the blinded data of the four patients in the block, we need to sum the conditional allocation probabilities of the first three allocation sequences. While for block size two, we used data from two patients to estimate the probabilities of two possible allocation sequences; for a block size of four, we used the data of four patients to estimate the probabilities of six possible allocations. In general, for block length *τ*, there are K=ττ/2 possible allocation sequences, each with unconditional probability 1/*K*, and we need to estimate *K* allocation probabilities based on the blinded data of *τ* patients. Because *K* >> *τ* for larger *τ*, it is intuitively clear that for larger block length the additional information provided by blocking decreases (see also 21).

To compute the worst case sample size reassessment rule in case of blocked randomization, we need to introduce some notation. Let T={1,1+τ,1+2τ,…,n−τ+1} denote the set of indices where a new block starts. For i∈T let $b_{i}=(x_{j},y_{j}){}_{j=i}^{i+\tau -1}$, denote the observations in the block starting with the *i* ^*th*^ patient. Let $\omega_{k}^{\left(i\right)}=(\omega_{k,j}^{\left(i\right)}){}_{j=1}^{\tau },k=1\ldots ,K$ denote the indicator vectors of the *K* possible treatment allocations for block $b_{i},i\in T$, where $\omega_{k,j}^{\left(i\right)}\in \{0,1\}$ and $\overset{\tau }{\underset{j=1}{\sum }}\omega_{k,j}^{\left(i\right)}=\tau /2$ for all i∈T. Here, $\omega_{k,j}^{\left(i\right)}=0$ denotes that in the *k* ^*th*^ treatment allocation for *i* ^*th*^ block the *j* ^*th*^ patient in the block was allocated to group A (control), and $\omega_{k,j}^{\left(i\right)}=1$ denotes that this patient was allocated to group B (treatment). Under block randomization, each allocation is equally likely, such that $P\left(\omega_{k}^{\left(i\right)}\right)=1/K$ for k=1,2,…,K and i∈T and the joint density for the observations *b*_*i*_ in block *i* is given by

$$f(b_{i})=\frac{1}{K}\overset{K}{\underset{k=1}{\sum }}f\left(b_{i}|\omega_{k}^{\left(i\right)}\right)\;,$$

where $f(b_{i}|\omega_{k}^{\left(i\right)})=\overset{\tau -1}{\underset{l=0}{\prod }}f(x_{i+l},y_{i+l}|g_{i+l}=\omega_{k,l+1}^{\left(i\right)})$, and $f(x_{i+l},y_{i+l}|g_{i+l}=\omega_{k,l+1}^{\left(i\right)})$ denotes a bivariate normal density with mean vector $\left(\nu_{0},\omega_{k,l+1}^{\left(i\right)}\nu_{1}+(1-\omega_{k,l+1}^{\left(i\right)})\nu_{0}\right)$, variances *σ* ^2^, and correlation *ρ*. Then, the conditional probability of each treatment allocation, given the data of block *b*_*i*_, is given by

$$P\left(\omega_{k}^{\left(i\right)}|b_{i}\right)=\frac{f\left(b_{i}|\omega_{k}^{\left(i\right)}\right)\cdot P(\omega_{k}^{\left(i\right)})}{f(b_{i})}=\frac{f\left(b_{i}|\omega_{k}^{\left(i\right)}\right)}{\overset{K}{\underset{k=1}{\sum }}f\left(b_{i}|\omega_{k}^{\left(i\right)}\right)}\;,\;k=1,2,\ldots ,K.$$

To derive the sample size reassessment rule that maximizes the type I error rate, we compute the conditional expectation and conditional variance of the first stage test statistics *Z*_1_, conditional on the blinded first stage observations $(X_{i},Y_{i}){}_{i=1}^{n_{1}}\;:$

$$m_{Z_{1}}=E\left(Z_{1}|(X_{i},Y_{i}){}_{i=1}^{n_{1}}=(x_{i},y_{i}){}_{i=1}^{n_{1}}\right)=\frac{1}{\sigma \sqrt{n_{1}}}\underset{i\in T}{\sum }E\left(m_{\tau ,i}|b_{i}\right)=\frac{\underset{i\in T}{\sum }\overset{K}{\underset{k=1}{\sum }}P\left(\omega_{k}^{\left(i\right)}|b_{i}\right)m_{\tau ,i}^{\left(k\right)}}{\sqrt{n_{1}}}\;,$$

$$v_{Z_{1}}=\mathrm{Var}\left(Z_{1}|(X_{i},Y_{i}){}_{i=1}^{n_{1}}=(x_{i},y_{i}){}_{i=1}^{n_{1}}\right)=\frac{1}{\sigma^{2}n_{1}}\underset{i\in T}{\sum }\left(\overset{K}{\underset{k=1}{\sum }}P\left(\omega_{k}^{\left(i\right)}|b_{i}\right)\left(m_{\tau ,i}^{\left(k\right)}\right){}^{2}-\left(\overset{K}{\underset{k=1}{\sum }}P\left(\omega_{k}^{\left(i\right)}|b_{i}\right)m_{\tau ,i}^{\left(k\right)}\right){}^{2}\right)\;,$$

where $m_{\tau ,i}=\overset{\tau -1}{\underset{l=0}{\sum }}(2G_{i+l}-1)X_{i+l}\;$, and $m_{\tau ,i}^{\left(k\right)}$ is a realization of *m*_*τ*,*i*_ at *k* ^*th*^ treatment allocation of *i* ^*th*^ block at (*X*_*i*_,*Y*_*i*_) = (*x*_*i*_,*y*_*i*_). As in the random allocation case, the conditional distribution of *Z*_2_ given the blinded first stage observations $(X_{i},Y_{i}){}_{i=1}^{n_{1}}$ is standard normal, and we approximate the conditional distribution of *Z*_1_ by a normal distribution with mean $m_{Z_{1}}$ and variance $v_{Z_{1}}$. As in the unblocked case, we can express the overall test statistic *Z*_*N*_ as a weighted sum of the stage wise test statistics such that the conditional error rate is given by

(9) $$P_{H_{0}}\left(Z_{N}>z_{1-\alpha }|(X_{i},Y_{i}){}_{i=1}^{n_{1}}=\left(x_{i},y_{i}\right){}_{i=1}^{n_{1}}\right)=1-\Phi \left(\frac{z_{1-\alpha }-\sqrt{\frac{n_{1}}{N}}m_{Z_{1}}}{\sqrt{\frac{n_{1}}{N}v_{Z_{1}}+\frac{n_{2}}{N}}}\right)\;.$$

If there are restrictions for the second stage sample size that is $n_{2}\in [n_{2}^{\min},n_{2}^{\max}]$ for some $n_{2}^{\min}$ and $n_{2}^{\max}$, then (Appendix A) the value of *n*_2_ maximizing (9) can be calculated as in the unblocked case by (7) or (8) with *m*_1_ and *V*_1_ replaced by $m_{Z_{1}}$ and $v_{Z_{1}}$, respectively.

**Figure 3:**
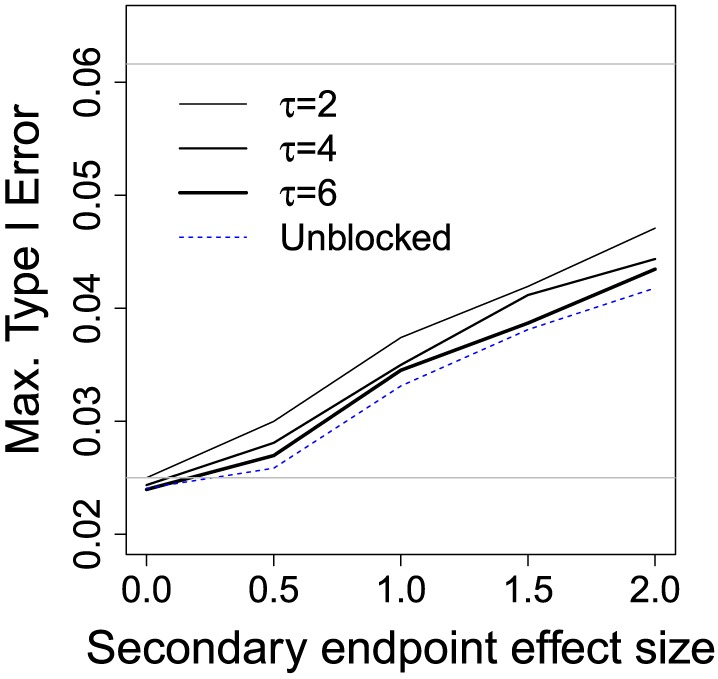
Maximum type I error rate without restrictions for the second stage sample size and for blocked randomization with block sizes 2, 4, 6 and the unblocked design (*n*_1_=144,*ρ* = 0,*σ* = 1, and 2.5·10^5^ (2·10^5^) simulation runs for block size 2 (4, 6, unblocked design)).

Figure 3 shows the maximum type I error rate of the trial with block randomization, with block sizes {2,4,6} (and per group sample size 72) for *ρ* = 0 and unrestricted second stage sample size (i.e., *n*_2_∈[0,+*∞*)). Results for other correlations for both unrestricted and restricted second stage sample size are given in the Supporting Information Figure 9.4. As expected, using the additional information on the blocking of observations increases the maximum type I error rate. The smaller the block size the better the data can be unblinded, and the larger is the maximal type I error rate.

To implement the aforementioned worst case sample size adaptation rule, one must know the block size. However, also if the block sizes are not known, the type I error rate may be inflated. Consider a clinical trial where block randomization is used, but the worst case sample size reassessment rule for random allocation (7), (8) is applied (which does not require knowledge of the block sizes). To estimate the type I error rate for such a setting, simulation studies for different block sizes were performed as in the preceding text. In all considered scenarios, the simulated maximum type I error rate is very close to the maximum error rate observed in the setting of Section 3, where the same sample size reassessment rule for random allocation (7), (8) is applied, but random allocation is used to allocate patients (Figure 9.7–9.10 in the Supporting Information).

## 5 A clinical trial example

As an illustrative example, consider a Phase III clinical trial to asses efficacy and safety of Fingolimod in patients with relapsing‐remitting multiple sclerosis along the lines of the FREEDOMS trial 22, 23. While in the original trial, 1,272 patients were randomized to receive oral Fingolimod doses of 0.5 or 1.25 mg or placebo daily; for simplicity, we consider a trial with two parallel groups, comparing only the higher dose with placebo with *N* = 800 patients in total, randomly allocated to groups (such that the per‐group sample size is similar to the original trial). The annualized aggregate relapse rate (ARR) during months 0 to end of study was set as a primary endpoint and is defined as the number of confirmed relapses in a year. Among the additionally measured parameters is the mean lymphocyte count. It is known from earlier studies that Fingolimod lowers the lymphocyte count compared wtih placebo. Based on the results in 24, we assume a mean *ν*_0_=1.8 [×10^9^ cells/L] for the placebo group and *ν*_1_=0.55[×10^9^ cells/L] for the 1.25 mg Fingolimod group with a common standard deviation of *σ*_*x*_=*σ*_*y*_=0.31[×10^9^ cells/L] for the mean lymphocyte counts at day 7 (values approximated based on Figure 6 in 24). Furthermore, it is known that the lymphocyte counts are causally related to the primary endpoint through the mechanism of action of Fingolimod. Lee JY *et al*. (2013)^25^ investigated the relation between the predicted lymphocyte count to ARR. Because no correlation coefficient is given in 25, and we do not have access to the raw data; we computed the maximum inflation of the type I error rate and the correlation between the blinded and unblinded effect size estimates for a grid of *ρ* in [0,0.9].

Assume that in the Phase III trial an interim analysis is performed after *n*_1_=400 subjects have been randomized. By (6), the maximum type I error rate for *ρ*∈[0,0.9] ranges from *α*_*max*_=0.054 to *α*_*max*_=0.059 and if we restrict the second stage sample size such that ${ñ}_{2}\in [200,1600]$ the maximum type I error rate ranges from *α*_*max*_=0.035 to *α*_*max*_=0.036 (see Supporting Information Figure 9.6 (a)).

As another example, assume that instead of the lymphocyte counts the mean total white blood cell counts (WBC) are used. The mean WBC count for the placebo group at 24 months is *ν*_0_=6.5 × 10^9^ cells/L with a standard deviation of 1.8, the mean for Fingolimod 1.25 mg group *ν*_1_=3.8 × 10^9^ cells/L with a standard deviation of 1.3 (estimated from the Supporting Information Figure 1 C in 26). As we are not aware of published data on the correlation of WBC and ARR, we computed the maximum inflation of the type I error rate and the correlation between the blinded and unblinded effect size estimates for a grid of *ρ* in [0,0.9]. Then, pooling the group wise standard deviations to *σ* = 1.57, the upper bound for the type I error rate for *ρ*∈[0,0.9] ranges from *α*_*max*_=0.041 to *α*_*max*_=0.054 for unrestricted second stage sample size and from *α*_*max*_=0.031 to *α*_*max*_=0.035 if the second stage sample size is restricted to the interval [200,1600] (see Supporting Information Figure 9.6 (b)).

## 6 Discussion

In this work, we demonstrated that even blinded sample size reassessment may lead to an inflation of the type I error rate if there are secondary endpoints for which the alternative holds. This implies that unscheduled sample size reassessment, even in a blinded setting, may damage the integrity of the trial. The numerical results give an upper bound for the inflation of the type I error that may occur due to blinded sample size reassessment in a setting where the distribution of a secondary endpoint is known to the experimenter, for example from historical data. While this is a simplifying assumption, the impact of a treatment on surrogate endpoints is often known from Phase II trials before a Phase III trial is started.

However, the approach can be extended to settings where no prior information on the distribution of the secondary endpoint is available. In this case, the distribution of the secondary endpoint can be estimated from the blinded data based on a mixture model with an expectation–maximization (EM) algorithm as in 27 or 28. It has been shown that such estimators, when applied to the data of the primary endpoint, are only reliable for very large effect or sample sizes 29 and perform poorly for effect sizes usually occurring in clinical trials. However, while large treatment effects in the primary endpoint do occur rarely, this does not necessarily apply to effect sizes for secondary or safety endpoints (see, for example, the clinical trial example in Section 5), which are relevant for the setting considered in this manuscript. Overall, depending on the effect size in the secondary endpoint, the type I error rate resulting from sample size reassessment based on expectation–maximization algorithms will still be affected, albeit on a lower scale.

In the computation of the worst case sample size reassessment rule, we used only the information from a single secondary endpoint to estimate the treatment allocation. Instead, one could use the data from several endpoints: to derive the resulting maximum type I error rate, one needs to replace the bivariate normal densities in (5) by the respective multivariate densities. To extend the setting of a single interim analysis to multiple blinded interim analyses, one can derive worst case adaptation rules and the resulting maximum type I error rate with a backwards induction approach.

We investigated the impact of different randomization procedures on the maximum type I error rate and found that block randomization, especially with small block sizes, increases the type I error rate inflation, if the information on the block size is used in the sample size adjustment. If the latter information is not used, blocking leads to essentially the same inflation as under random allocation. These findings support current recommendations against too small block sizes and inclusion of information on block sizes in study protocols 30.

Wang *et al*. 31 consider a related problem and derive the maximum type I error rate for sample size reassessment rules based on *unblinded* interim effect size estimates of a secondary endpoint that is correlated with the primary endpoint, but assuming that the primary endpoint is not observed in the interim analysis. The maximum type I error rate in this setting depends only on the correlation *ρ* of the primary and the secondary endpoints, and there is no inflation of the type I error rate if *ρ* = 0. In contrast, in the blinded setting considered in this paper, even if the correlation between the primary and the secondary endpoint is zero, the type I error rate may be inflated. This holds because we assume that the primary endpoint is observed and the blinding is partially lost due to a treatment effect in the secondary endpoint that gives information on the treatment allocation. The potential inflation of the type I rate is related to the fact that this partial loss of blinding allows one to estimate the unblinded first stage effect size estimate in the primary endpoint $\bar{X}=[\overset{n_{1}}{\underset{i=1}{\sum }}2(2G_{i}-1)X_{i}]/n_{1}$: if the unknown *G*_*i*_ are replaced by *q*_*i*_ as defined in (5), a blinded estimate is given by ${\bar{X}}_{b}=[\overset{n_{1}}{\underset{i=1}{\sum }}2(2q_{i}-1)X_{i}]/n_{1}\;$. The correlation *r* between $\bar{X}$ and ${\bar{X}}_{b}$ (not to be confused with the correlation *ρ* between primary and secondary endpoint) can be interpreted as a measure of unblinding and increases with the effect size in the secondary endpoint and *ρ*. In the clinical trial of Section 5, for example, *r* ranges from 0.97 up to nearly 1 in the first and from 0.68 to 0.96 in the second example for *ρ*∈[0,0.9] (see Figure 9.5 in the Supporting Information Figures and Section 8.1 in the Supporting Information for computational details).

Our findings do not contradict the well established use of blinded sample size reassessment based on aggregate event rates or variance estimates computed from blinded primary endpoint interim data. However, they demonstrate that the type I error rate control of these methods relies on the application of specific, binding, pre‐planned, and fully algorithmic sample size reassessment rules (as recommended for data monitoring committee charters, see for example 32) for which type I error control has been demonstrated. The type I error rate control does not extend to general sample size adjustments based on blinded data. Therefore, including only a non‐binding option for blinded sample size reassessment in clinical trial protocols is not sufficient to guarantee type I error rate control. In particular, we quantify the maximum type I error rate inflation when a worst case adaptation rule is applied that also uses information from a secondary endpoint.

Our work also implies that post hoc adjustments of the sample size may lead to type I error rate inflations, even if justified by post hoc scientific arguments (as required in the guideline quoted in the Introduction). Consider, for example, a scenario where blinded outcome data is available and adaptations following the rule in Section 3.3 are applied whenever a post hoc selected sample size reassessment rule (or scientific arguments external to the trial) can be found that justifies that choice. Otherwise, the pre‐specified sample size is used. Because the conditional error rate is increased in all instances where the sample size is adapted but is unchanged otherwise, the overall type I error rate will be inflated by such a strategy. Furthermore, note that even aggregate statistics (as referred to in the quoted guidelines) may contain information on the unblinded treatment effect estimate and therefore may lead to type I error rate inflation. Examples are the correlation coefficient of the primary endpoint and a secondary or safety endpoint (if there is a treatment effect in the latter), or per group means of subgroups whose definition is based on such secondary or safety endpoints. While the assumption that a worst case sample size rule is applied in an actual clinical trial may not be realistic, it is a means to derive an upper bound for the type I error rate in settings where no binding sample size reassessment procedure is pre‐specified, or post hoc adaptations are performed, and secondary endpoint data has been available. While the actual type I error may be substantially lower than this upper bound, it can not be computed because it depends not only on the realized sample sizes but also on the sample sizes that would have been applied had other interim data been observed.

In settings where no sample size adjustment algorithm has been pre‐specified, alternatives to fixed sample hypothesis tests are tests based on combination functions or the conditional error rate principle 2, 4, 33, 34 that control the type I error rate even without pre‐specified adaptation rules. The conditional error rate based procedures even control the type I error rate if no adaptations were pre‐planned but are introduced during the conduct of the study.

## Supporting information

Supporting info item

## Appendix A Computation of the sample size reassessment rule maximizing the type I error rate.

### A.1

To maximize the type I error rate in the second stage sample size ${ñ}_{2}$, one needs to maximize the corresponding conditional error rate, which is (6) for the random allocation case and (9) for the blocked case. For computational convenience, let us express both conditional error rates as functions of $R=\frac{{ñ}_{2}}{n_{1}}$. Then, in both considered cases, the conditional error rate can be expressed as 1 − *Φ*(*f*(*R*)), where

$$f(R)=\frac{z_{1-\alpha }\sqrt{1+R}-m}{\sqrt{v+R}}\;$$

with *m* = *m*_1_ and *v* = *V*_1_ for (6) and with $m=m_{Z_{1}}$ and $v=v_{Z_{1}}$ for (9). Finding the ${ñ}_{2}$ that maximizes the conditional error rate is then equivalent to determining the *R* that minimizes *f*. The latter can be found by taking the derivative of *f* in *R*. We consider the general case where the second stage sample size may be restricted that is ${ñ}_{2}\in [n_{2}^{\min},n_{2}^{\max}]$ for some $n_{2}^{\min}\geq 0$ and $n_{2}^{\max}\leq \infty$. This translates to boundaries *R*_*m**i**n*_,*R*_*m**a**x*_ for *R* where $R_{\min}=n_{2}^{\min}/n_{1}$ and $R_{\max}=n_{2}^{\max}/n_{1}$. In the unrestricted case, we set $n_{2}^{\min}=0$ and $n_{2}^{\max}=\infty$ such that *R*_*m**i**n*_=0 and *R*_*m**a**x*_=*∞*. The first derivative of *f* is given by

$$\frac{\mathrm{\partial f}(R)}{\mathrm{\partial R}}=\frac{z_{1-\alpha }(v-1)+m\sqrt{1+R}}{2\sqrt{1+R}(v+R){}^{3/2}}\;.$$

Assume first that *v* < 1. To determine extrema of *f*, we consider the following cases:

1. *m*≤0. Then, $\frac{\mathrm{\partial f}(R)}{\mathrm{\partial R}}<0$ and *f*(*R*) is minimized at $\tilde{R}=R_{\max}$;
2. $m\in \left(0,\frac{z_{1-\alpha }(1-v)}{\sqrt{1+R_{\max}}}\right)$. Then,
  $$\frac{\mathrm{\partial f}(R)}{\mathrm{\partial R}}<\frac{z_{1-\alpha }(1-v)\left[\sqrt{\frac{1+R}{1+R_{\max}}}-1\right]}{2\sqrt{1+R}(v+R){}^{3/2}}\;,$$
  and because $\sqrt{\frac{1+R}{1+R_{\max}}}\leq 1,\frac{\mathrm{\partial f}(R)}{\mathrm{\partial R}}<0$ for *R*∈[*R*_*m**i**n*_,*R*_*m**a**x*_] and *f*(*R*) is minimized at $\tilde{R}=R_{\max}$;
3. $m\in \left[\frac{z_{1-\alpha }(1-v)}{\sqrt{1+R_{\max}}},\frac{z_{1-\alpha }(1-v)}{\sqrt{1+R_{\min}}}\right]$.
  Then, $\frac{\mathrm{\partial f}(R)}{\mathrm{\partial R}}=0$ for
  $$\tilde{R}=\left(\frac{z_{1-\alpha }(1-v)}{m}\right){}^{2}-1\;;$$
  $\tilde{R}$ is indeed a local minimum because the second derivative of *f*
  $$\frac{z_{1-\alpha }(1-v)(3+v+4R)-3m(1+R){}^{3/2}}{4(1+R){}^{3/2}(v+R){}^{5/2}}\;,$$
  evaluated at $\tilde{R}$ is equal to
  $$-\frac{m^{6}(v-1)}{4z_{1-\alpha }^{2}\left((v-1){}^{3}\left(m^{2}+(v-1)z_{1-\alpha }^{2}\right)\right){}^{3/2}}>0\;.$$
  Furthermore,
  $$f(\tilde{R})=\sqrt{\frac{z_{1-\alpha }^{2}(v-1)+m^{2}}{v-1}}\;.$$
4. $m>\frac{z_{1-\alpha }(1-v)}{\sqrt{1+R_{\min}}}$. Then,
  $$\frac{\mathrm{\partial f}(R)}{\mathrm{\partial R}}>\frac{z_{1-\alpha }(1-v)\left[\sqrt{\frac{1+R}{1+R_{\min}}}-1\right]}{2\sqrt{1+R}(v+R){}^{3/2}}\;,$$
  and because $\sqrt{\frac{1+R}{1+R_{\min}}}>1,\frac{\mathrm{\partial f}(R)}{\mathrm{\partial R}}>0$ for *R*∈[*R*_*m**i**n*_,*R*_*m**a**x*_] and *f*(*R*) is minimized at $\tilde{R}=R_{\min}$.

Taking all the four cases together, we obtain for *v* < 1 that the $\tilde{R}$ minimizing *f*(*R*) is

(A.1) $$\tilde{R}(m,v)=\left\{\begin{array}{cc} R_{\max} & \text{if}m<z_{*}\\ \left[\left(\frac{z_{1-\alpha }(1-v)}{m}\right){}^{2}-1\right] & \text{if}m\in \left[z_{*},z^{*}\right]\\ R_{\min} & \text{if}m>z^{*} \end{array}\right.,$$

where $z_{*}=\frac{z_{1-\alpha }(1-v)}{\sqrt{1+n_{2}^{\max}/n_{1}}},z^{*}=\frac{z_{1-\alpha }(1-v)}{\sqrt{1+n_{2}^{\min}/n_{1}}}$.

Now, taking ${ñ}_{2}(m,v)=n_{1}\tilde{R}$, we obtain the sample size reassessment rule (for *v* < 1)

(A.2) $${ñ}_{2}(m,v)=\left\{\begin{array}{cc} n_{2}^{\max} & \text{if}m<z_{*}\\ \left[\left(\frac{z_{1-\alpha }(1-v)}{m}\right){}^{2}-1\right]n_{1} & \text{if}m\in \left[z_{*},z^{*}\right]\\ n_{2}^{\min} & \text{if}m>z^{*} \end{array}\right.,$$

If now *v* > 1, then consider the following cases:

- *m*≥0 : then, $\frac{\mathrm{\partial f}(R)}{\mathrm{\partial R}}>0$ and *f*(*R*) is minimized at $\tilde{R}=R_{\min}$,
- *m*∈(*z* ^∗^,*z*_∗_): then, $\frac{\mathrm{\partial f}(R)}{\mathrm{\partial R}}>0$ and *f*(*R*) is minimized at $\tilde{R}=R_{\min}$,
- *m*≤*z* ^∗^ : then, *f*(*R*) is minimized at $\tilde{R}=\left(\frac{z_{1-\alpha }(1-v)}{m}\right){}^{2}-1$,
- *m* > *z*_∗_: then, $\frac{\mathrm{\partial f}(R)}{\mathrm{\partial R}}>0$ and *f*(*R*) is minimized at $\tilde{R}=R_{\min}$.

Summarizing for *v* > 1 the $\tilde{R}$ minimizing *f*(*R*) is

(A.3) $$\tilde{R}(m,v)=\left\{\begin{array}{cc} R_{\min} & \text{if}m>z^{*}\\ \left[\left(\frac{z_{1-\alpha }(1-v)}{m}\right){}^{2}-1\right] & \text{if}m\leq z^{*} \end{array}\right.,$$

Taking ${ñ}_{2}(m,v)=n_{1}\tilde{R}$, we obtain the sample size reassessment rule (for *v* > 1)

(A.4) $${ñ}_{2}(m,v)=\left\{\begin{array}{cc} n_{2}^{\min} & \text{if}m>z^{*}\\ \left[\left(\frac{z_{1-\alpha }(1-v)}{m}\right){}^{2}-1\right]n_{1} & \text{if}m\leq z^{*} \end{array}\right..$$
