# Supplementary material for: Maximum type I error rate inflation from sample size reassessment when investigators are blind to treatment labels
Source: Stat Med. 2015 Dec 23;35(12):1972–84. doi: 10.1002/sim.6848 (PMC4851240; doi:10.1002/sim.6848)
Supplement: Supplementary file 1 — Supporting info item [file SIM-35-1972-s001.pdf]

8. Supplementary Material

8.1. Approximation for correlation between blinded and unblinded effect size estimates

We prove that the correlation r between blinded and unblinded effect size estimates $\bar{X}_b$ and $\bar{X}$, defined in the Discussion is

$$r = \sqrt{\frac{E(q_1 X_1^2 | G_1 = 1) - \sigma^2/2}{\sigma^2/2}} ,$$

where

$$E(q_1 X_1^2 | G_1 = 1) = \int_{\boldsymbol{R}^2} \frac{x^2 \varphi^2_{\nu_1,\sigma,\rho}(x,y)}{\varphi_{\nu_0,\sigma,\rho}(x,y) + \varphi_{\nu_1,\sigma,\rho}(x,y)} d(x,y) .$$

Proof:
Without loss of generality, assume, that $\nu_0 = 0$. Observe first, that

$$E(\bar{X}) = E\left(\frac{2\sum_{i=1}^{n_1}(2G_i - 1)X_i}{n_1}\right) = \frac{\sum_{i=1}^{n_1}[4E(G_i X_i) - 2E(X_i)]}{n_1} = 0$$

$$E(\bar{X}_b) = E\left(\frac{2\sum_{i=1}^{n_1}(2q_i - 1)X_i}{n_1}\right) = \frac{\sum_{i=1}^{n_1}[4E(q_i X_i) - 2E(X_i)]}{n_1} = 0,$$

since

$$E(q_i X_i) = \frac{1}{2}\int x_i \varphi_{\nu_1,\sigma,\rho}(x_i, y_i) d(x_i, y_i) = \frac{1}{2} E(X | G = 1) = 0 .$$

Furthermore

$$E(\bar{X}\bar{X}_b) = \frac{\sum_{i=1}^{n_1}\left[E(q_i X_i^2 | G_i = 1) - \sigma^2/2\right]}{{n_1}^2/8} ,$$

and

$$Var(\bar{X}) = E(\bar{X}^2) = \frac{E\left(\left(2\sum_{i=1}^{n_1}(2G_i - 1)X_i\right)^2\right)}{{n_1}^2} = \frac{4\sigma^2}{n_1} .$$

Furthermore

$$Var(\bar{X}_b) = E(\bar{X}_b^2) = \frac{\sum_{i=1}^{n_1}\left[E\left(q_i^2 X_i^2\right) - E(q_i X_i^2) + E(X_i^2)/4\right]}{{n_1}^2/16} ,$$

and having

$$E(q_i X_i^2) = \frac{1}{2}\int x_i^2 \varphi_{\nu_1,\sigma,\rho}(x_i, y_i) d(x_i, y_i) = \frac{1}{2} E(X_1^2 | G_1 = 1) = \frac{\sigma^2}{2} ,$$

Copyright © 0000 John Wiley & Sons, Ltd.

and

$$E(q_i^2 X_i^2) \quad = \quad \frac{1}{2}\int x_i^2 q_i \varphi_{\nu_1,\sigma,\rho}(x_i, y_i) d(x_i, y_i) = \frac{1}{2} E(q_1 X_1^2 | G_1 = 1) \ ,$$

one gets

$$Var(\bar{X}_b) \quad = \quad \frac{\sum_{i=1}^{n_1}\left[\frac{1}{2}E\left(q_i X_i^2 | G_i = 1\right) - \frac{\sigma^2}{4}\right]}{{n_1}^2/16} = \frac{E(q_1 X_1^2|G_1 = 1) - \sigma^2/2}{n_1/8} \ .$$

Then,

$$r \quad = \quad \frac{\sqrt{E(q_1 X_1^2|G_1 = 1) - \sigma^2/2}}{\sqrt{\sigma^2/2}} \ ,$$

where

$$E(q_1 X_1^2|G_1 = 1) \quad = \quad \int_{\boldsymbol{R}^2} \frac{x^2 \varphi_{\nu_1,\sigma,\rho}^2(x, y)}{\varphi_{\nu_0,\sigma,\rho}(x, y) + \varphi_{\nu_1,\sigma,\rho}(x, y)} d(x, y) \ .$$

For the numerical calculations we computed this integral with the `adaptIntegrate` function in the R-package `cubature`.

 Copyright © 0000 John Wiley & Sons, Ltd.

9. Supplementary figures

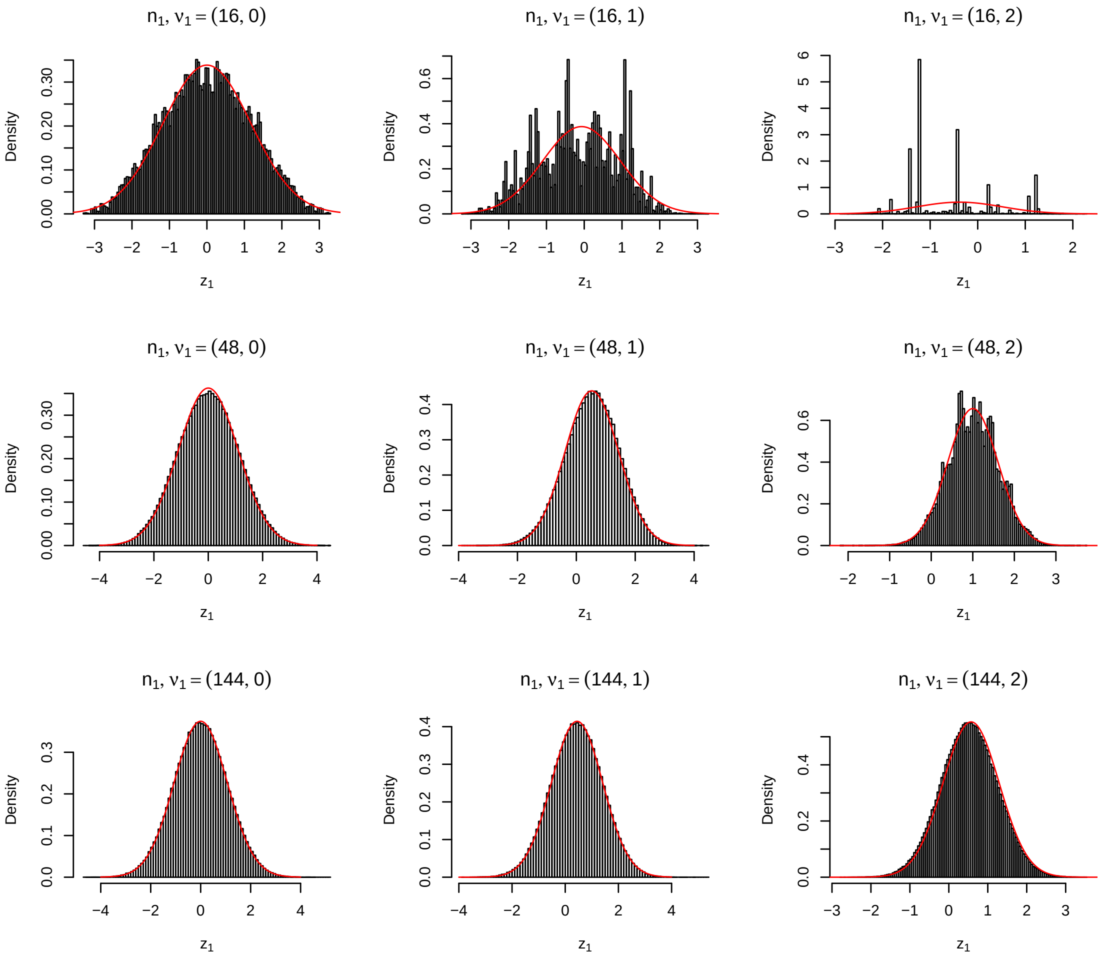

Figure 9.1. Histograms of draws from the density of $Z_1 \mid (X_i, Y_i)_{i=1}^{n_1}$ with normal approximation curve superimposed for various choices of n_1 and ν_1 and $\rho = 0$.

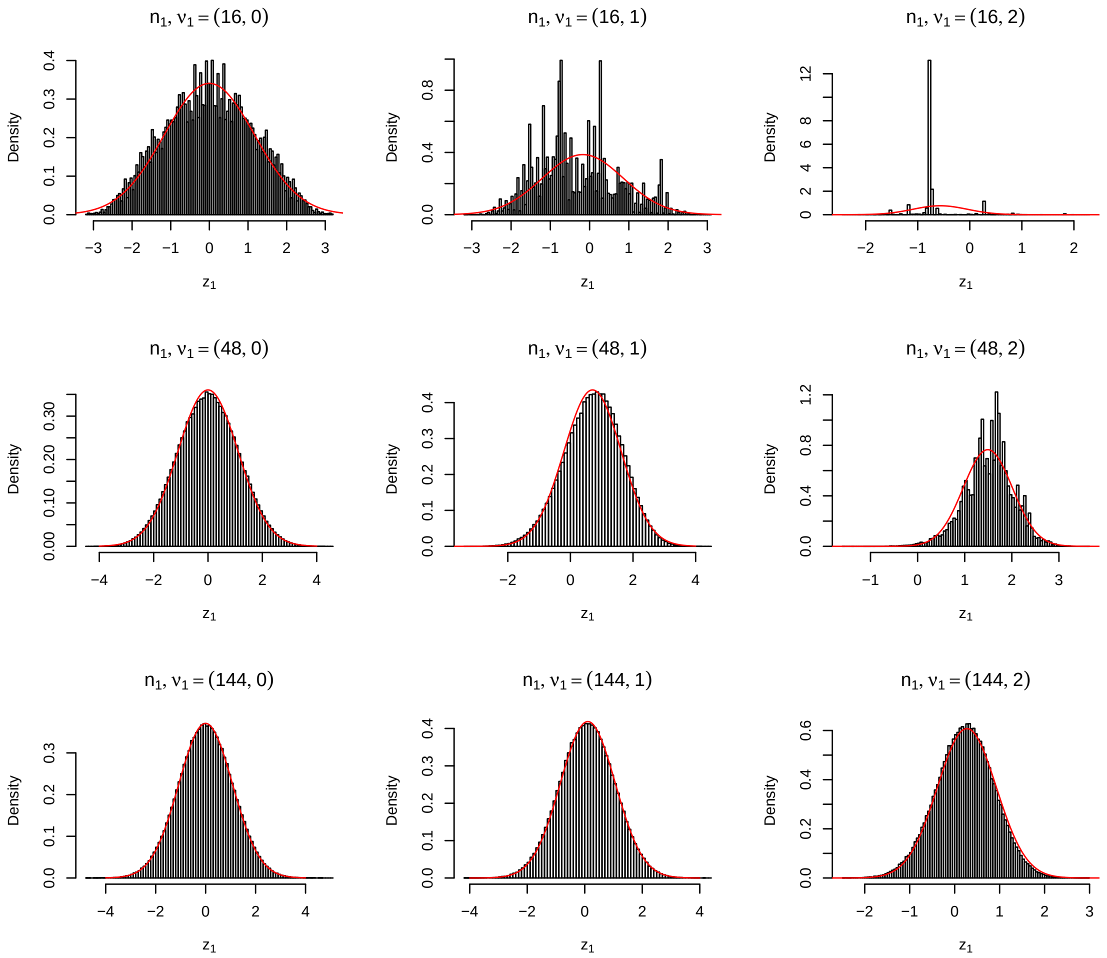

Figure 9.2. Histograms of draws from the density of $Z_1 \mid (X_i, Y_i)_{i=1}^{n_1}$ with normal approximation curve superimposed for various choices of n_1 and ν_1 and $\rho = 0.5$.

Copyright © 0000 John Wiley & Sons, Ltd.

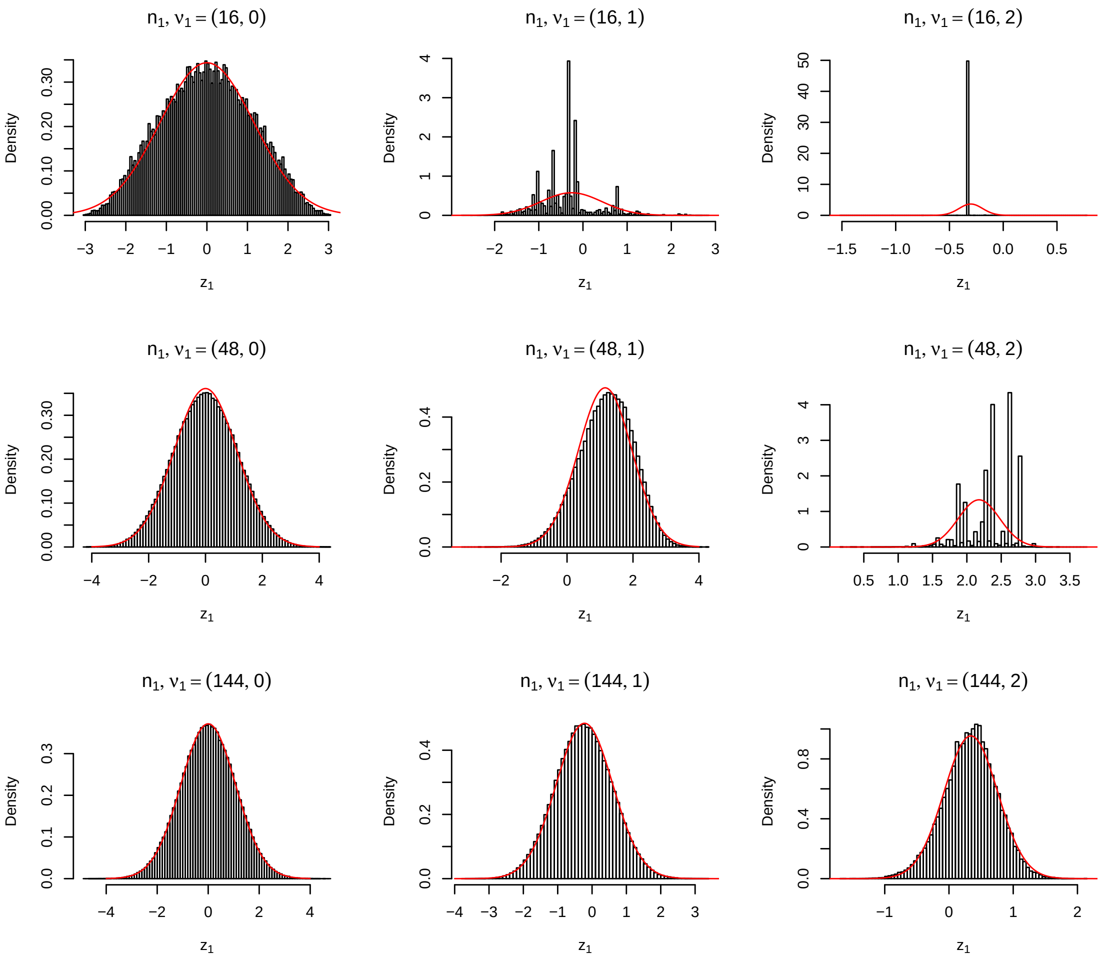

Figure 9.3. Histograms of draws from the density of $Z_1 \mid (X_i, Y_i)_{i=1}^{n_1}$ with normal approximation curve superimposed for various choices of n_1 and ν_1 and $\rho = 0.8$.

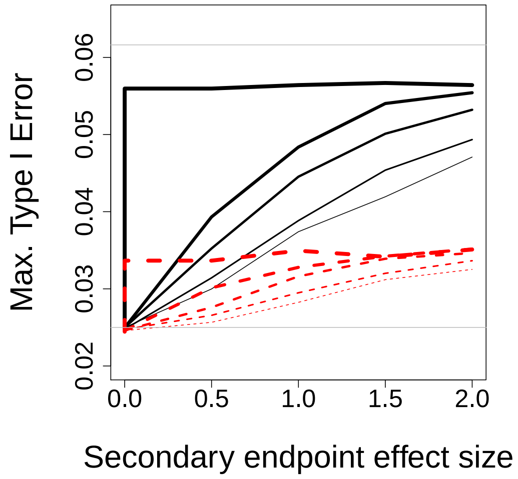

Figure 9.4. Maximum type I error rate for blocked randomization with block size 2, $n_1 = 144$, $\sigma = 1$ and correlations $\rho \in \{0, 0.5, 0.8, 0.9, 1\}$ (from bottom to top). Black solid lines denote unrestricted results and red dashed lines results for restricted case with $n_2^{min} = n_1/2$ and $n_2^{max} = 4n_1$. $\rho \in \{0, 0.5, 0.8, 0.9, 1\}$ and the larger the ρ the thicker the line.

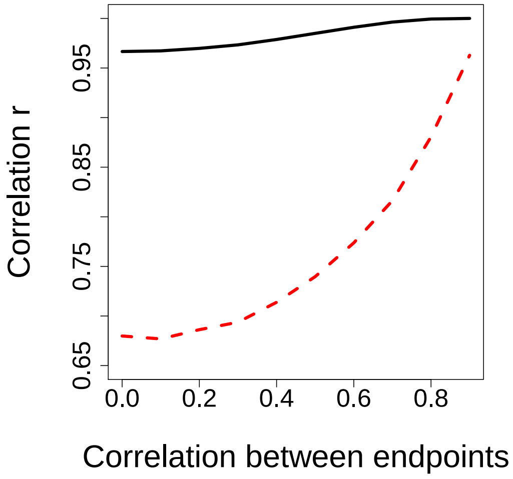

Figure 9.5. Correlation r between blinded and unblinded effect size estimates as a function of the correlation ρ between primary and secondary endpoint for the clinical trial examples in Section 5. Results are given for $n_1 = 400$ and $2 \cdot 10^5$ simulation runs with $\nu_1 = 1.25$ and $\sigma = 0.31$ for example 1 (black solid line) and $\nu_1 = 2.7$, $\sigma = 1.57$ for example 2 (red dashed line).

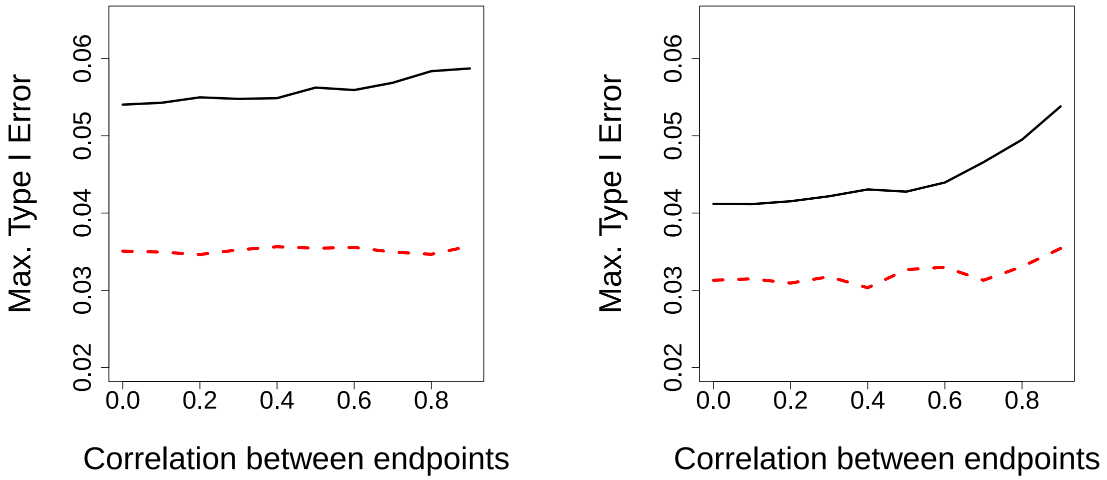

(a) **(b)**

Figure 9.6. Maximum type I error rate as a function of the correlation between endpoints for the two clinical trial examples with and without restrictions for the second stage sample size. Results obtained for $n_1 = 400$ and $2 \cdot 10^5$ simulation runs. Solid (dashed) lines give the type I error rate for unrestricted (restricted, with $n_2^{min} = n_1/2$ and $n_2^{max} = 4n_1$) sample size adjustment. (a) Example 1. ($\nu_1 = 1.25$, $\sigma = 0.31$), (b) Example 2. ($\nu_1 = 2.7$, $\sigma = 1.57$).

 Copyright © 0000 John Wiley & Sons, Ltd.

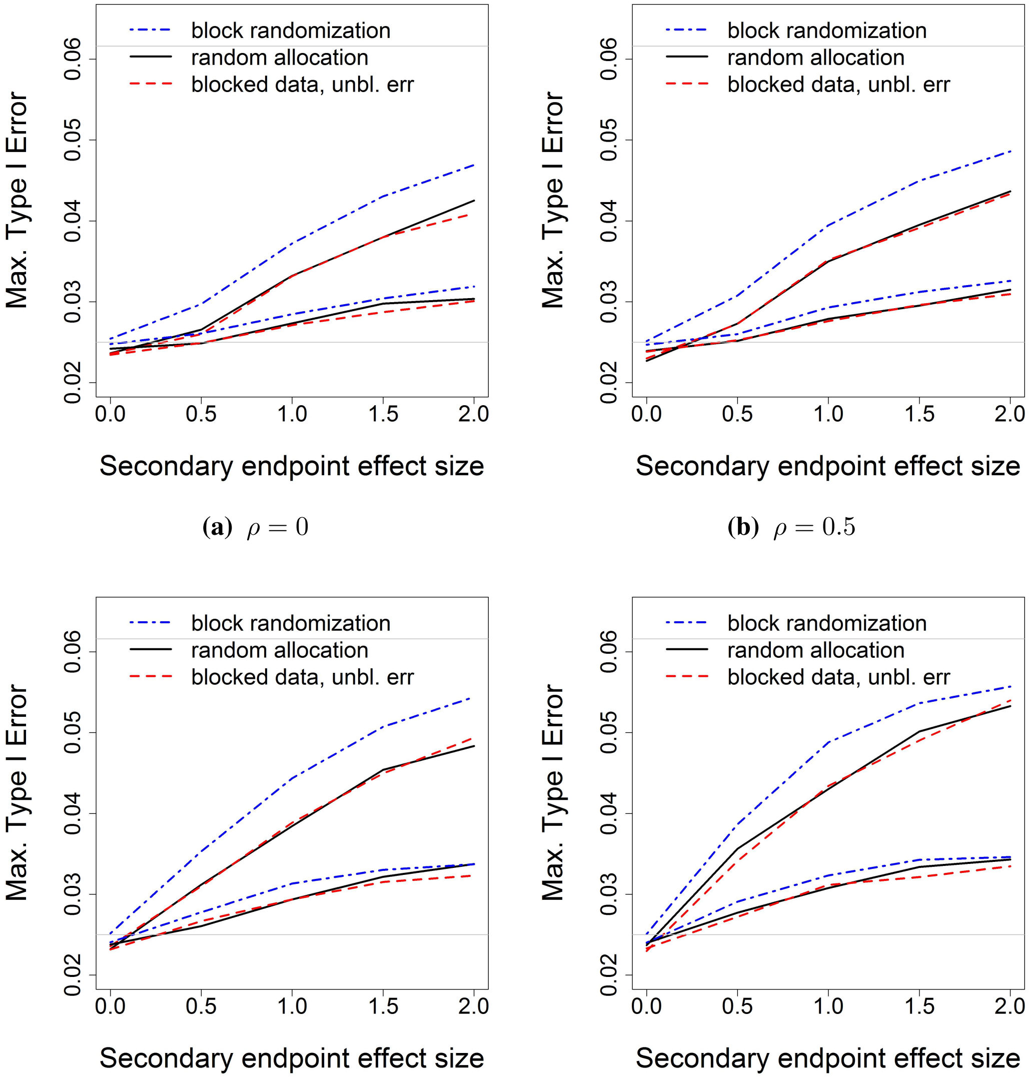

(a) $\rho = 0$ **(b)** $\rho = 0.5$

(c) $\rho = 0.8$ **(d)** $\rho = 0.9$

Figure 9.7. Maximum type I error rate as a function of the secondary endpoint effect size without restrictions for the second stage sample size (three upper lines) and with restrictions $n_2^{min} = n_1/2$ and $n_2^{max} = 4n_1$. Here the block size for block randomization was $\tau = 2$, first stage sample size $n_1 = 144$ and $\sigma = 1$ and $2.5 \cdot 10^5$ simulation runs. Except from the block randomization (blue dotted lines) and random allocation (black solid lines), the hypothetical scenario was considered, where the data were simulated based on the block randomization, but the type I error rate was calculated as under the random allocation (red dotted lines).

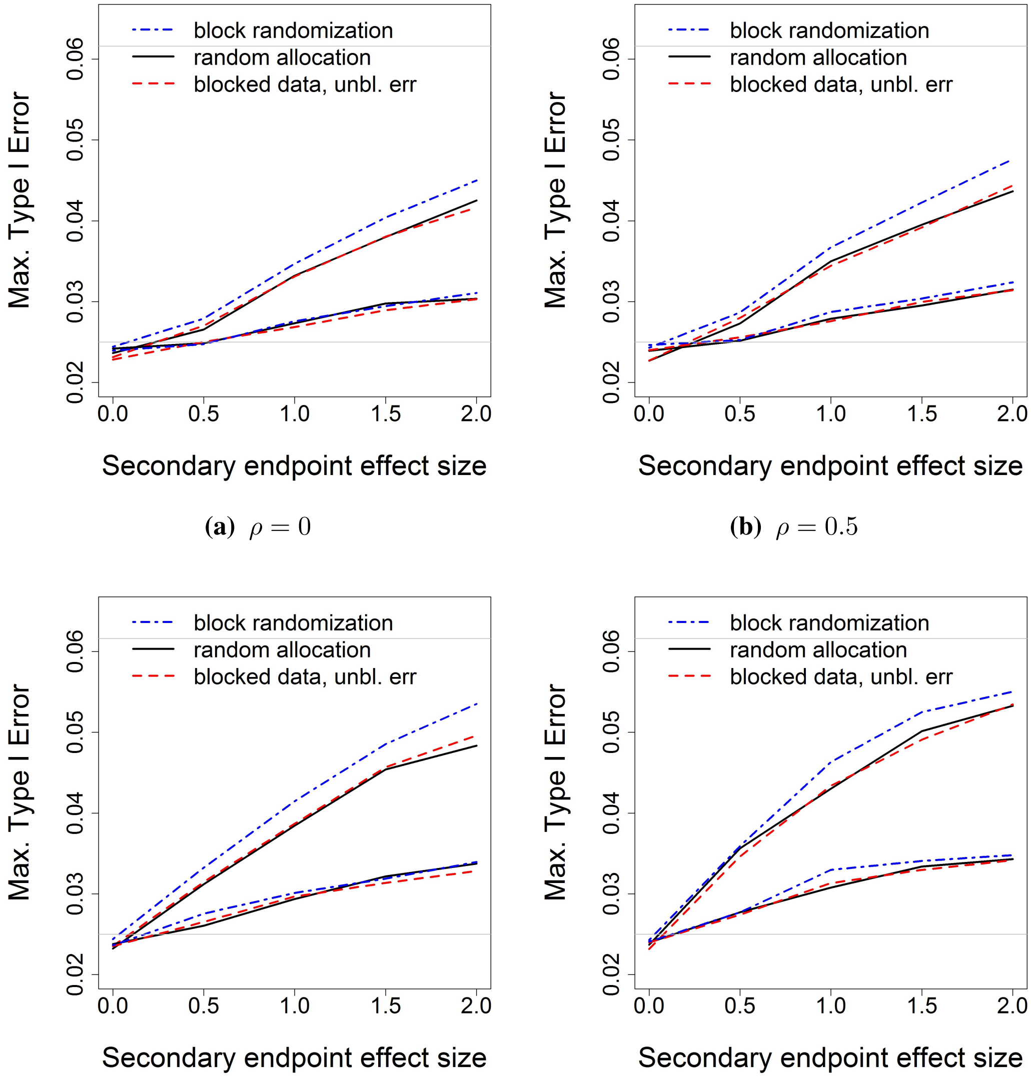

(a) $\rho = 0$ **(b)** $\rho = 0.5$

(c) $\rho = 0.8$ **(d)** $\rho = 0.9$

Figure 9.8. Maximum type I error rate as a function of the secondary endpoint effect size without restrictions for the second stage sample size (three upper lines) and with restrictions $n_2^{min} = n_1/2$ and $n_2^{max} = 4n_1$. Here the block size for block randomization was $\tau = 4$, first stage sample size $n_1 = 144$ and $\sigma = 1$ and $2.5 \cdot 10^5$ simulation runs. Except from the block randomization (blue dotted lines) and random allocation (black solid lines), the hypothetical scenario was considered, where the data were simulated based on the block randomization, but the type I error rate was calculated as under the random allocation (red dotted lines).

 Copyright © 0000 John Wiley & Sons, Ltd.
Prepared using simauth.cls

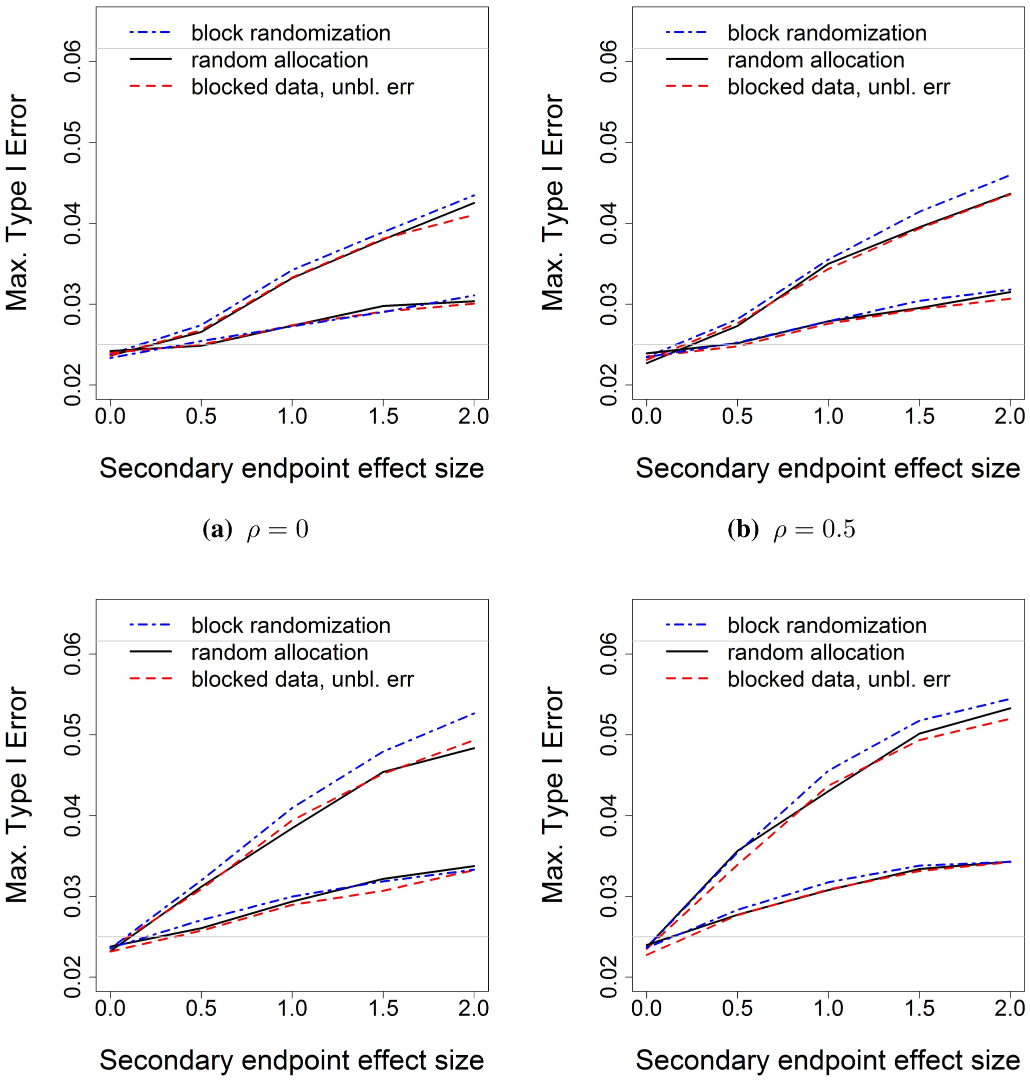

(a) $\rho = 0$ (b) $\rho = 0.5$

(c) $\rho = 0.8$ (d) $\rho = 0.9$

Figure 9.9. Maximum type I error rate as a function of the secondary endpoint effect size without restrictions for the second stage sample size (three upper lines) and with restrictions $n_2^{min} = n_1/2$ and $n_2^{max} = 4n_1$. Here the block size for block randomization was $\tau = 6$, first stage sample size $n_1 = 144$, $\sigma = 1$ and $2.5 \cdot 10^5$ simulation runs. Except from the block randomization (blue dotted lines) and random allocation (black solid lines), the hypothetical scenario was considered, where the data were simulated based on the block randomization, but the type I error rate was calculated as under the random allocation (red dotted lines).

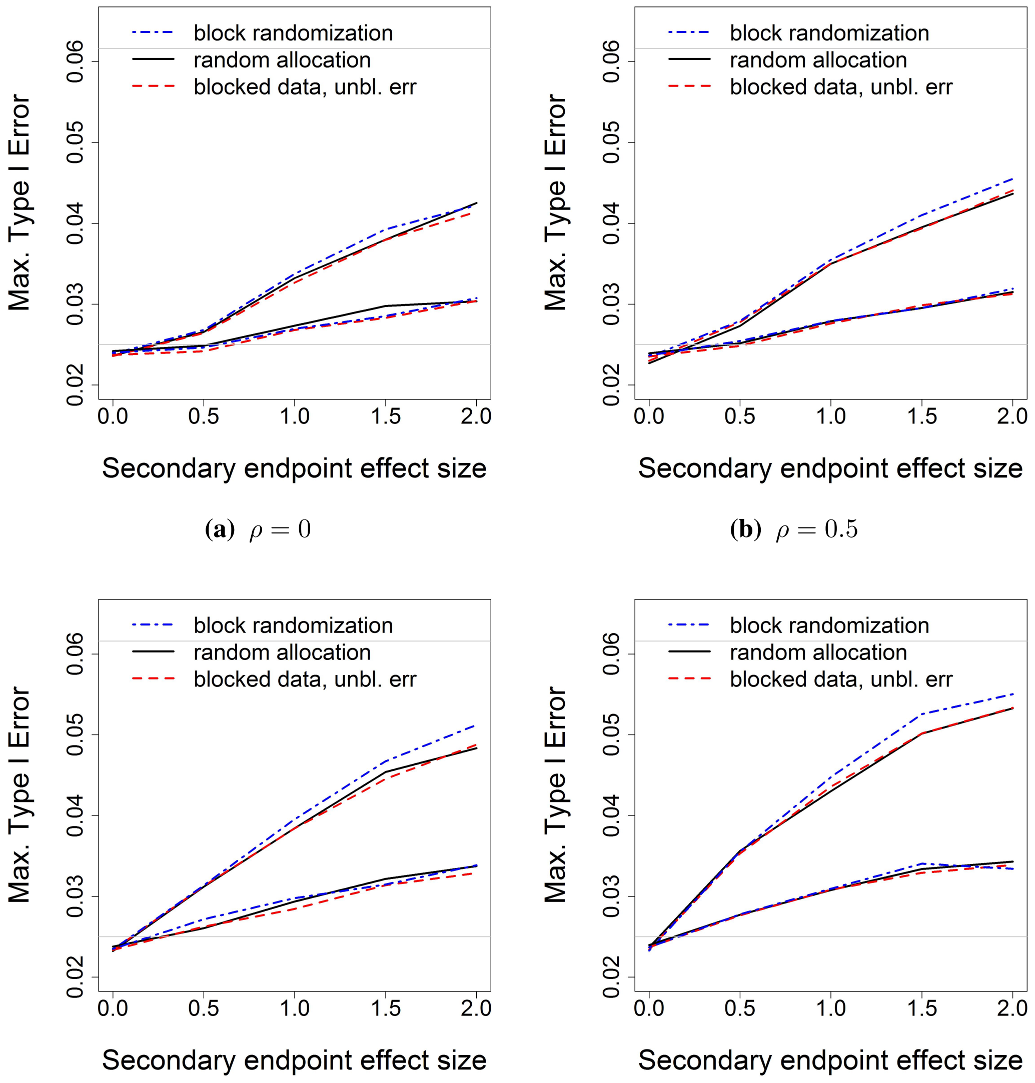

(a) $\rho = 0$ **(b)** $\rho = 0.5$

(c) $\rho = 0.8$ **(d)** $\rho = 0.9$

Figure 9.10. Maximum type I error rate as a function of the secondary endpoint effect size without restrictions for the second stage sample size (three upper lines) and with restrictions $n_2^{min} = n_1/2$ and $n_2^{max} = 4n_1$. Here the block size for block randomization was $\tau = 8$, first stage sample size $n_1 = 144$, $\sigma = 1$ and $2.5 \cdot 10^5$ simulation runs. Except from the block randomization (blue dotted lines) and random allocation (black solid lines), the hypothetical scenario was considered, where the data were simulated based on the block randomization, but the type I error rate was calculated as under the random allocation (red dotted lines).

 Copyright © 0000 John Wiley & Sons, Ltd.
